# Deep margin elevation; Indications and periodontal considerations

**DOI:** 10.34172/japid.2024.024

**Published:** 2024-11-06

**Authors:** Parnian Alizadeh Oskoee, Sana Dibazar

**Affiliations:** ^1^Department of Esthetic and Restorative Dentistry, Faculty of Dentistry, Tabriz University of Medical Sciences, Tabriz, Iran; ^2^Department of Esthetic and Restorative Dentistry, Faculty of Dentistry, Tabriz Medical Sciences, Islamic Azad University, Tabriz, Iran

## Dear Editor,

 Supragingival margins are preferred in restorative dentistry to maintain a healthy periodontium. However, some clinical situations, such as extensive carious lesions, esthetic demands, pre-existing deep margin restorations, or the need for retention, lead to subgingival margins.^1^ Localized subgingival margins can complicate adhesive restorations because of biological (biologic width violation and gingival inflammation) and operative problems (isolation, impression taking, and delivery) that hinder their durability and relationship with the periodontal tissues.^2-4^ In other words, the subgingival margins are difficult for clinicians to manage because of cavity preparation, caries removal, impression taking, isolation, overhanging problems, and the biological width (BW) violation probability.^5^

 High biofilm accumulation,^6^ gingival bleeding, increased probing depth, loss of periodontal attachment, and increased gingival recession probability are reported to be more common with subgingival restorations compared to supragingival restorations.^7^ In addition, Newcomb^8^ concluded a positive relation between gingival inflammation severity and marginal proximity to epithelial attachment. Regarding periodontal health, BW is the most important component.^9^ Typically, BW is compromised during the restoration of severe cervical defects, resulting in periodontal problems, gingival recession or inflammation, decreased bone level, and bleeding.^10^

 Achieving continued and optimal oral health requires a symbiotic interaction between gingival health and restorations.^9^ Maintaining periodontal health and supra-crestal tissue attachment necessitates the presence of ideal restorations around subgingival restorations.^11^

 Various clinical approaches are used to deal with subgingival restoration challenges.^12^ The conventional approaches include surgical crown lengthening (SCL) and orthodontic extrusion (OE) or a combination of both techniques, leading to an apical displacement of supporting tissues to access the subgingival margin and obtain adequate space to establish BW. The techniques mentioned above are associated with drawbacks like further attachment loss, exposure of root concavities and furcations, opening proximal contacts, black triangles, papilla atrophy, dentin hypersensitivity, patient discomfort, unfavorable crown-to-root ratio, compromised esthetics, and increased treatment time and cost.^9^

 The primary objective of modern restorative dentistry favors minimally invasive preparatory approaches and protocols.^13^ Dietschi and Spreafico’s 1998 proposal of “deep margin elevation” (DME) is a helpful non-invasive alternative for cavity margin displacement concerning BW. DME was the new term Magne and Spreafico gave it after it was initially known as coronal margin displacement.^12^ This minimally invasive restorative technique offers the possibility of performing stepwise elevation of localized deep proximal cavities using composite resin restorations. This approach focuses on the local isolation of deep margins using a modified circumferential metal matrix to create more favorable margins for direct, semi-direct, and indirect restorations. DME addresses multiple clinical problems associated with subgingival margins due to limited access (impression taking, both digitally and traditionally, bonding procedure, cementation, and excess removal), rubber dam slippage over the margin, and subsequent saliva, crevice fluid, and blood leakage.^12,14^ The absence of a recovery period is the most attractive advantage of the procedure compared to SCL.^4^ Additionally, DME and immediate dentin sealing can be used together to improve indirect adhesive restorations’ bond and marginal seal, correct geometry, reinforce undermined cusps, and fill undercuts.^4,15^

 Despite the apparent advantages of this technique, there are situations such as connective compartment violation of supra-crestal attachment, inability to isolate the field with a rubber dam, and inability to place the matrix that hinders the practice of DME.^16^ One of the main limitations of this technique is when the floor of the cavity is located within connective tissue or too close to the bone crest. The margin elevation requires a higher distance of 2 mm.^4^

 Different patterns of supra-crestal attachment may be observed upon the subgingival placement of a composite resin base. The long junctional epithelium is the only way to acquire periodontal attachment because histological analysis revealed no connective tissue connection.^17^ The periodontium’s capacity to produce a different, healthy BW with a longer junctional epithelium and a smaller connective tissue attachment beneath the material employed has been demonstrated as its tolerance of DME.^18^ Moreover, it has been noted that epithelial fibers might adhere to the resin restorations’ surface.^19^ Therefore, the only method for achieving periodontal adhesion to the substance is through the long junctional epithelium.^17^

 When evaluating the effectiveness and success of the DME technique, periodontal health must be assessed regarding bleeding on probing (BOP) and marginal bone level through radiographs.^2^ According to clinical/histological investigations in humans, DME and subgingival restorations are compatible with periodontal health if they are well-polished and refined, BW is not breached, and strict supportive periodontal care with recall and gingival inflammation monitoring along with appropriate oral hygiene is followed.^20,21^ In the case of margins positioned 2 mm from the bone crest, a substantial incidence of BOP is expected despite the low gingival index and plaque index rate.^2^ However, the extent of BW violation may determine the biological reaction of hard and soft tissues.^22^ For instance, infringement of a small proximal area is better tolerated than a whole circumferential margin under strict oral hygiene guidelines. A randomized clinical study found that after six months, subgingival proximal restorations impinging BW similarly produced identical plaque index, probing depth, and BOP with SCL groups under a strict plaque control program.^23^

 Ghezzi et al^19^ demonstrated that if the field can be isolated with a rubber dam, no surgical management is necessary, regardless of the depth of the lesion. However, the gingival biotype significantly impacts periodontal healing surrounding subgingival restorations. There is a correlation between gingival thickness and treatment outcomes, suggesting that thicker biotypes may be able to tolerate DME better and encounter fewer problems.^17^

 Finally, it can be mentioned that DME is a promising technique that conservatively relocates the cervical margins and is a useful option, especially for patients who cannot afford more invasive procedures. Although DME has a better survival rate, SCL is advised for the best chance of long-term success in cases where deep margins extend 2.0 mm beyond the bone crest.^2^ Furthermore, DME is a technique-sensitive treatment that needs to be used carefully, with respect to three criteria: the matrix’s perfect seal of the cervical border, the field isolation capability, and no invasion of the connective tissue within the BW.^15^ As current evidence is not enough to encourage the use of this technique with predictable outcomes, randomized clinical trials with extended follow-up periods are necessary to determine the technique’s validity in clinical practice and elucidate all aspects of it.

## Competing Interests

 The authors declare no competing interests in the publication of their work, either financial or non-financial.

## Ethical Approval

 Not applicable.
